# The Glycopeptide PV-PS A1 Immunogen Elicits Both CD4+ and CD8+ Responses

**DOI:** 10.3390/vaccines12121375

**Published:** 2024-12-06

**Authors:** Sharmeen Nishat, Md Kamal Hossain, Geraud Valentin, Farzana Hossain, Shanika Gamage, Katherine A. Wall, Peter R. Andreana

**Affiliations:** 1 Department of Chemistry, Bangladesh University of Engineering and Technology (BUET), Dhaka 1000, Bangladesh; 2 Department of Medicinal and Biological Chemistry, College of Pharmacy and Pharmaceutical Sciences, The University of Toledo, 2801 West Bancroft Street, Toledo, OH 43606, USAkatherine.wall@utoledo.edu (K.A.W.); 3 Department of Chemistry and Biochemistry and School of Green Chemistry and Engineering, University of Toledo, 2801 West Bancroft Street, Toledo, OH 43606, USA

**Keywords:** PS A1, cancer immunotherapy, MHCI, MHCII, conjugate vaccine, SPPS, Thomsen nouveau, CD8+

## Abstract

Background/Objectives: The MHCII-dependent, CD4+ T-cell zwitterionic polysaccharide PS A1 has been investigated as a promising carrier for vaccine development because it can induce an MHCII-dependent CD4+ response towards a variety of tumor-associated carbohydrate antigens (TACAs). However, PS A1 cannot elicit cytotoxic T lymphocytes through MHCI, which may or may not hamper its potential clinical use in cancer, infectious and viral vaccine development. This paper addresses PS A1 MHCI independence through the introduction of an MHCI epitope, the poliovirus (PV) peptide, to establish an MHCI- and MHCII-dependent vaccine. Methods: We synthesized a glycopeptide construct targeting the Thomsen-nouveau TACA (Tn-PV-PS A1) and a control Tn-PV peptide. C57BL/6 mice were immunized with both constructs, and the resulting T-cells were extracted from spleens. Results: Through cell proliferation assays, we show that Tn-PV-PS A1 elicits a robust CD4+ and CD8+ immune response. The resulting cytotoxic T lymphocytes are specific towards Tn-PV and trigger cell lysis of Tn-expressing EL4 cells. Conclusions: This study confirms PV-PS A1 as a robust MHCI- and MHCII-dependent carrier. This is the first report of MHCI dependence in a zwitterionic polysaccharide.

## 1. Introduction

It has long been known that only proteins/peptides can be processed and presented to T-cells by the MHCII system. As a result, most carbohydrates are considered T-cell-independent and only elicit a weak IgM response. To overcome this shortcoming, carbohydrate antigens have historically been conjugated to strongly immunogenic protein carriers [1]. This approach has proven successful against infectious disease, and conjugate vaccines are currently approved in the US against meningitis [2] and pneumonia [3], amongst others. Consequently, tumor-specific antigens, present on the surface of virtually all cancer cells, have drawn considerable attention as potential targets for immunotherapy. Among these tumor-associated carbohydrate antigens (TACAs), the ones expressed on mucin MUC1, including abnormally truncated glycans such as Thomsen-nouveau (Tn), Thomsen–Friedenreich (TF) and their sialylated derivatives, have garnered the most attention, ranking second on the NCI priority list of cancer antigens [4] due to MUC1’s high immunogenicity, role in immune evasion, and abundant expression in lung [5], breast [6], colon [7] or prostate [8] epithelial adenocarcinomas [9,10]. However, the challenging tumor microenvironment and the numerous tumor immune suppression mechanisms have, until now, prevented any conjugate vaccine from completing phase III clinical trials for immunotherapy [11,12].

One reason for this shortcoming lies in the protein carriers themselves; their very high immunogenicity diverts the immune response away from the target antigen, a phenomenon known as epitope suppression [13]. A new generation of carriers has emerged to assist in developing better alternatives, including virus-like particles [14], nanoparticles [15], and most recently, zwitterionic polysaccharides (ZPSs) [16]. The latter are a class of unusually immunogenic carbohydrates. Their structure contains alternating positive charges on adjacent monosaccharide units, which gives them an α-helical structure mimicking proteins and enabling presentation by the MHCII pathway similarly to protein antigens [17]. Our group has studied PS A1, a 110 kDa ZPS from *Bacteroides fragilis* (ATCC 25285/NCTC) 9343 capsule, as a possible vaccine carrier and demonstrated its efficacy to elicit strong CD4+ responses towards a variety of TACAs [16]. Notably, a Tn-PS A1 construct targeting the Tn antigen elicited a strong antigen-specific response, with low epitope suppression [18]. The vaccine construct was also proven to elicit class-switching to IgGs, and triggered complement-dependent cytotoxicity (CDC) and natural killer-mediated antibody-dependent cell cytotoxicity (ADCC) [19].

MHCII restriction, however, might be a drawback to PS A1’s allure as an immunotherapeutic carrier, as MHCI-dependent cytotoxic T lymphocyte activation has been noted for a durable immune response against cancer [20]. In this work, we introduce a new, modified PS A1 that includes a poliovirus (PV)-derived MHCI epitope, establishing PS A1 as a polyvalent, MHCI- and MHCII-dependent carrier for immunotherapy development. The PV peptide, KLFAVWKITYKDT, is the 103–115 fragment of the capsid protein VP1 from poliovirus type 1 Mahoney strain [21]. While the peptide has been extensively used in TACA-targeting vaccines as a T-helper MHCII epitope [22,23,24], the VP1 protein is known to bind to MHCI and MHCII H2 molecules, the murine equivalent of HLA [25,26]. Two MHCI H2-K^d^ epitopes have been proposed by Chow et al. for VP1 [25]. One, TYKDTVQLRR (Chow peptide; 110–120 fragment), overlaps with the PV peptide. This prompted us to assess the PV peptide for MHCI binding. To our surprise, not only did it bind better, in silico, to H2-K^d^ than the Chow peptide, but the PV peptide can also bind to H2-K^b^, H2-D^b^ and a large selection of human HLAs, reducing the risk of potential late-stage clinical failure. The large body of data supporting PV peptide safety and compatibility with TACA vaccines, and its predicted MHCI dependency, could make it a viable MHCI epitope for PS A1-based vaccines. In this study, we prove that the inclusion of the PV peptide to develop a Tn-PV-PS A1 (I) vaccine elicits both CD4+ and CD8+ responses in C57BL/6 mice, cementing PV as a key polyvalent epitope for vaccine design, and PV-PS A1 as a robust MHCII- and MHCI-dependent carrier for immunotherapy development.

## 2. Materials and Methods

**Statistical Analysis.** Mice group sizes were calculated based on the amount of serum and cells required for analysis. Statistical analysis of all assays was performed using Prism 10 (GraphPad, Boston, MA, USA). Each assay was analyzed by one-way ANOVA. Finer pairwise comparisons within the assays were calculated using Student’s *t*-test. A *p*-value of 0.05 was considered significant.

**Figure 1:**
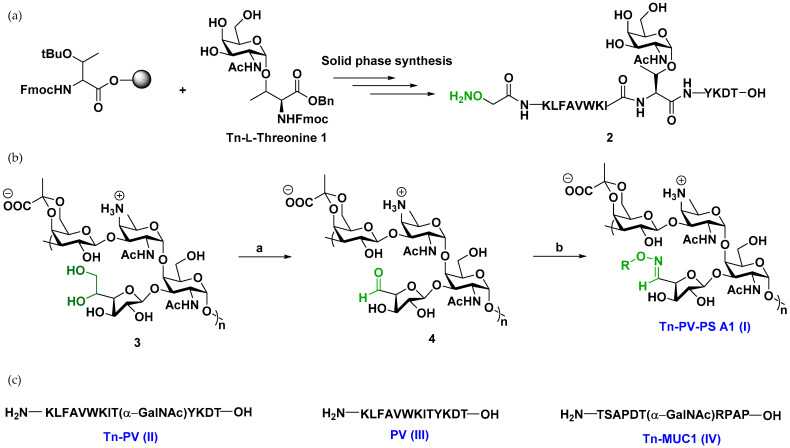
(**a**) Solid phase peptide synthesis (SPPS) of Tn-PV-ONH_2_. (**b**) Synthesis of Tn-PV-PS A1 (I) a. 100 mM acetate buffer (pH 5.0), 1 mM NaIO_4_ solution, dark, rt, 1 h; b. **2**, 100 mM acetate buffer, 37 °C, overnight. (**c**) Other targets synthesized by Solid Phase Peptide Synthesis.

**Mice Immunizations.** All research was approved by the Institutional Animal Care and Use Committee of the University of Toledo (IACUC protocol UT-400102). Mice were obtained from the Jackson laboratory (JAX). Six- to seven-week-old C57BL/6 (expressing H2-K^b^ and H2-D^b^) mice were allowed to acclimate for 1 week prior to intraperitoneal (ip) immunizations. Mice were divided into three subgroups consisting of five mice in each group. Group A mice were immunized with vaccine construct (I) (Figure 1), group B mice were immunized with Tn-PV (II) (Figure 1) and group C mice were injected with PBS as a control. Constructs I and II were dissolved in 1X PBS buffer (pH 7.4) at a concentration of 1 mg/mL. Mice were inoculated on day 0 and boosted three times (days 14, 28, 42) with 100 µL of emulsion (50 µg construct in 50 µL PBS + 50 µL TiterMax^®^ Gold). On day 49, mice were sacrificed, and serum and spleens were collected and pooled from each group of mice. Serum was stored at −80 °C for further studies and the spleens were immediately processed to isolate CD4+ and CD8+ T-cells.

**Culture of Bone Marrow-Derived Dendritic Cells (DCs).** Femur and tibia bones were collected from non-immunized C57BL/6 mice and muscles were removed using a blade [27]. The bones were sterilized by dipping them in 70% ethanol for 1 min followed by washing with ice-cold PBS in a Petri dish to remove ethanol. Finally, the bones were placed in 5 mL ice-cold T-cell medium [28] in a sterile Petri dish and bone marrow was collected by cutting the two ends of the bones and flushing with T-cell medium using a syringe and 25 G needle. The bone marrow suspension was transferred to a sterile 15 mL round-bottomed tube and centrifuged at 800 rcf for 5 min. The supernatant was discarded, and the pellet was resuspended in 1 mL red blood cell (RBC) lysing buffer Hybri-Max^TM^ (Sigma Aldrich, St. Louis, MO, USA). After one minute, the lysis buffer was quenched with 10 mL T-cell medium and filtered through a 0.70 µm filter. The filtrate was centrifuged at 800 rcf for 5 min, the supernatant was discarded, and the cells were suspended in ice-cold T-cell medium to a concentration of 10^6^ cells/mL. Granulocyte macrophage-colony stimulating factor (GM-CSF, PeproTech^®^, Cranbery, NJ, USA) at 100 U/mL (10 ng/mL) and Interleukin-4 (IL-4, PeproTech^®^) at 10 ng/mL were added to the medium and the culture was incubated at 37 °C for 3 days. On day 3, 75% of the cell suspension was removed and centrifuged at 600 rcf for 5 min. The supernatant was removed, the cell pellet resuspended in fresh T-cell medium and then transferred back to the old flask followed by the addition of GM-CSF and IL-4 each at 10 ng/mL concentration. After 3 days of incubation at 37 °C, DCs were centrifuged at 600 rcf for 5 min, suspended in T-cell medium and kept on ice for the next use.

**T-cell Separation.** Isolated spleens were placed in a freshly prepared T-cell culture medium and homogenized to prepare a spleen cell suspension. The cell suspension was transferred to a 15 mL sterile tube, centrifuged, and the supernatant was removed. The pellet was incubated for two minutes with RBC lysing buffer. A total of 10 mL of T-cell media was added to the cells and the suspension was filtered through a 70 µm filter. The cells were then washed three times with T-cell culture media by centrifuging the cells at 400 rcf for 5 min and removing supernatant. Finally, CD4+ and CD8+ T-cells were isolated from the spleen cell suspension using a Dynabeads^®^ FlowComp™ Mouse CD4 and CD8 kit, respectively (Invitrogen™, Naugatuck, CT, USA), following manufacturer guidelines.

**CD8+ T-cell Proliferation Assay.** Seventy-five microliters of irradiated DC suspension cultured from the bone marrow of a non-immunized C57BL/6 mouse were added to each well (2 × 10^4^ cells/well) in a flat-bottomed 96-well plate and pulsed with the antigens by incubating with 50 µL of Tn-PV (II) solution for 30 min at room temperature at antigen concentrations of 0, 10, 20 and 40 µg/mL. Seventy-five-microliter aliquots of isolated CD8+ T-cell suspension (2 × 10^5^ cells/well) from group A mice were added to the well. Therefore, the total volume in each well was 200 µL and the DC-to-CD8+ ratio was 1:10. In some wells, 2 µg/mL concanavalin A was substituted for antigen as a positive control. The plate was incubated at 37 °C in the presence of 5% CO_2_ for 4 days. On day 4, [^3^H] thymidine (40 µCi/mL, 25 µL/well, Moravek Chemicals, Brea, CA, USA) was added to each well and the plate was incubated overnight. On the following day, cells were harvested on a glass fiber membrane filter and the plate radioactivity was read on a Top Count scintillation counter to determine the thymidine incorporation for each group. Another proliferation assay was performed by pulsing irradiated DCs with 10 µg/mL Tn-PV (II) or PV (III) (Figure 1) or PBS to compare antigen-specific CD8+ T-cell proliferation of separate groups of mice following the same protocol. Counts per minute (CPM) values obtained from separate groups were compared to evaluate Tn antigen-specific proliferation of CD8+ T-cells.

**CD4+ T-cell Proliferation Assay.** A total of 75 µL of irradiated DC suspension was added to each well (2 × 10^4^ cells/well) of a flat-bottomed 96-well plate and pulsed with the antigens by incubating with 50 µL of Tn-PV (II) solution for 30 min at room temperature at antigen concentrations of 0, 10, 20, 40 and 80 µg/mL. Seventy-five-microliter aliquots of isolated CD4+ cell suspension (2 × 10^5^ cells/well) from group A mice were added to the well. Therefore, the total volume in each well was 200 µL and the DC-to-CD4+ ratio was 1:10. ConA was added to the well containing non-pulsed DCs (2 × 10^4^ cells/well) and CD4+ cells (2 × 10^5^ cells/well) isolated from group A mice as positive control. The plate was incubated at 37 °C in the presence of 5% CO_2_ for 4 days. On day 4, [^3^H] thymidine was added to each well (40 µCi/mL, 25 µL/well) and the plate was incubated overnight. On the following day, cells were harvested using a cell harvester instrument on a glass fiber membrane Whatman^®^ UNIFILTER^®^ 96-well filter plate clear polystyrene, GF/B membrane (Perkin Elmer, Waltham, MA, USA) and the plate was read on a Top Count scintillation counter (Packard, Downers Grove, IL, USA) to determine the thymidine incorporation. CPM values obtained from different concentrations of Tn-PV (II) were compared to evaluate the antigen-specific proliferation of CD4+ cells. Another proliferation assay was performed by pulsing irradiated DCs with 40 µg/mL Tn-PV (II) to compare antigen-specific CD4+ T-cell proliferation of separate groups of mice following the same protocol.

**Interferon Gamma (IFN-γ) Production.** DC suspension cultured from a non-immunized C57BL/6 mouse was plated in a 24 well-plate (500 µL, 2 × 10^4^ cells/well). DCs were pulsed with 30 µL of PV (III) or Tn-PV (II) (25 µg/mL final concentration) and unpulsed DCs were kept as negative control. CD8+ T-cells (2 × 10^5^ cells/well) isolated from mice (group A and group B) were added to the wells containing unpulsed or pulsed DCs. The total volume in each well was 1 mL and the DC-to-CD8+ T-cell ratio was 1:10. The plate was incubated at 37 °C for 24 h at 5% CO_2_ and the supernatant was collected and stored at −70 °C for ELISA analysis. Released IFN-γ was measured with a murine IFN-γ Mini ELISA Development Kit (PeproTech^®^).

**JAM Apoptosis Assay with Peptide Pulsed and Unpulsed EL4 Cells.** The assay is based on a published procedure [29] where C57BL/6 EL4 lymphoma cells (ATCC^®^ TIB-39™) are freshly cultured in DMEM medium supplemented with 10% FCS and the cell concentration brought to 1 × 10^5^ cells/mL. Therefore, 5 mL of EL4 cell suspension was incubated overnight in medium only (control) or medium supplemented with 30 µg/mL CD8+ T-cell epitope Tn-MUC1 (IV). The next day, [^3^H] thymidine (5 µCi/mL) was added separately to both epitope-pulsed and unpulsed EL4 suspension and incubated for 5 h. In the meantime, the CD8+ T-cells from each group were isolated as described above and the concentrations of the cells were brought to 1.25 × 10^6^ cells/mL and 2.50 × 10^6^ cells/mL. Afterwards, [^3^H] thymidine-incorporated EL4 cells were washed with growth medium at 400 rcf for 5 min to remove any residual [^3^H] thymidine. The cell concentration was brought to 5 × 10^4^ cells/mL and a 100 µL aliquot was added to each well of a U-bottom 96-well plate (5 × 10^3^ cells/well). One hundred-microliter aliquots of prepared CD8+ T-cell suspensions (1.25 × 10^6^ cells/mL and 2.50 × 10^6^ cells/mL) were added separately to different wells containing peptide pulsed and unpulsed EL4 so that the EL4-to-CD8+ ratio was 1:25 or 1:50. A total of 100 µL of 2 µM staurosporine (positive control) or 100 µL EL4 growth media (negative control) was added to some wells containing peptide pulsed or unpulsed EL4, i.e., in the absence of CD8+ T-cells. The plate was incubated for 6 h at 37 °C at 5% CO_2_. The cells were then harvested on a glass fiber membrane Unifilter-96 GF/B plate (Perkin Elmer, Waltham, MA, USA) and [^3^H] thymidine incorporation was determined on a Top Count scintillation counter (Packard, Downers Grove, IL, USA). Cytotoxicity was calculated using the following formula:

% Cytotoxicity 1 − E/S × 100

% Specific cytotoxicity = % cytotoxicity of peptide-pulsed EL4 − % cytotoxicity of unpulsed EL4

where S is the CPM value from wells with EL4 only and E is the CPM value from wells with EL4 and CD8+ T-cells.

## 3. Results and Discussion

**Computational analysis of MHCI epitopes.** Binding capabilities of VP1 protein fragments to MHCI H2-K^d^ were screened using the consensus/SMM method on the immune epitope database (IEDB) to evaluate the IC50 of potential murine MHCI epitopes in the KLFAVWKITYKDT (103–115, PV peptide (III)) and TYKDTVQLRR (110–120, Chow) peptides [30,31]. This method uses a database of known MHCI epitopes to evaluate the contribution of every amino acid at every position of the binding groove to MHCI binding affinity. The process is repeated with amino acid pairs. The resulting scoring table is used to evaluate prospective epitopes. Only values <5000 nM were kept (Table 1A). As anticipated, the Chow peptide binds moderately well to H2-K^d^. This is consistent with VP1’s ability to elicit cytotoxic T lymphocytes (CTLs) in H2-K^d^-expressing BALB/c mice. The adjacent PV peptide (III) also showed significant binding. This could indicate that the sequence they share, TYKDT, is part of the key H2-K^d^ MHCI epitope. While all binding values are fairly high (>5000 nM is considered a non-binder), glycosylation is not considered, and is known to improve binding [32,33]. Addition of the Tn antigen in the vaccine has the potential to increase binding compared to the algorithmic predictions. Encouraged by this finding, we evaluated whether either peptide could be used in different mice strains, or in humans. To do this, we simulated binding to murine MHCI H2-K^b^ and H2-D^b^, and to a sample of 40 common alleles of human HLAs to evaluate long-term potential in human drugs. The PV peptide (III) demonstrated superiority in both; it was the sole peptide capable of binding to both H2-K^d^ and H2-K^b^ (Table 1) and it is predicted to bind with great affinity to a broad variety of human HLAs (three best epitopes). Based on these results, the PV peptide (III) is best tested in b haplotype mice strains like C57BL/6, where numerous epitopes from its sequence are predicted to bind both H2-K and H2-D. The lower IC50s predicted for human HLA binding suggests that these results will translate well in clinical trials.

**Vaccine synthesis.** Synthesis of the vaccine construct commenced with Tn-L-threonine antigen **1** by established literature procedures [34] (Scheme S1). **1** was incorporated into the fully deprotected glycopeptide **2** (Tn-PV-ONH_2_) by solid phase peptide synthesis (SPPS) (Figure 1, Scheme S2). Selective oxidation of PS A1 with sodium metaperiodate (NaIO_4_) gave PS A1 aldehyde, which was subsequently treated with **2** at 37 °C overnight at pH 5 (Scheme S1) to afford Tn-PV-PS A1 conjugate (I). The success of the conjugation was confirmed by a distinct oxime doublet at δ 7.55 on ^1^H NMR. Antigen loading was determined to be ~10% using NMR by comparing the oxime hydrogen at 7.55 ppm to the PS A1 NHAc peaks at 2.0 ppm. Compound **1** was also used as a precursor to synthesize glycopeptides Tn-PV conjugate (II) and Tn-MUC1 (IV) by SPPS (Scheme S2).

**Figure 2:**
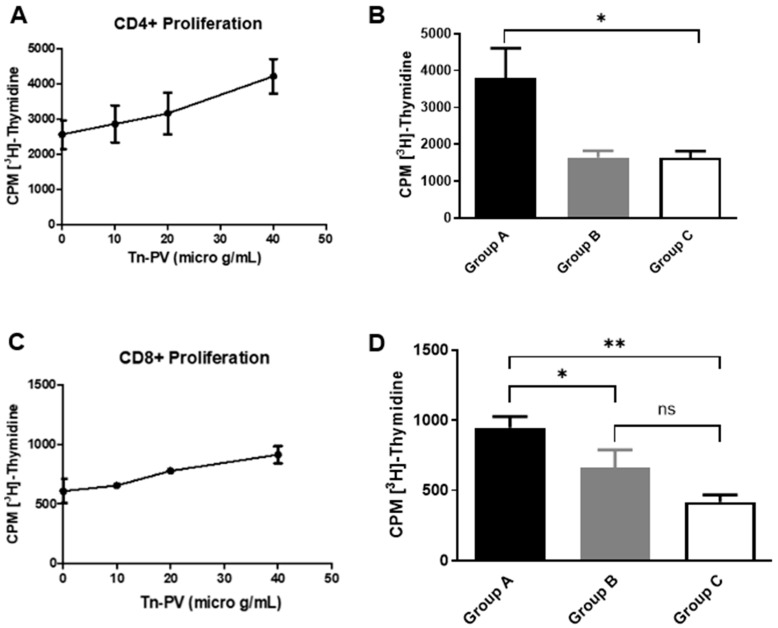
T-cell proliferation assays. (**A**) Proliferation of Group A CD4+ T-cells at different Tn-PV (II) concentrations. (**B**) Proliferation of CD4+ T-cells from all mice stimulated with 40 µg/mL Tn-PV (II). (**C**) Proliferation of Group A CD8+ T-cells at different Tn-PV (II) concentrations. (**D**) Proliferation of CD8+ T-cells from all mice with 10 µg/mL Tn-PV (II). * *p*-value < 0.05, ** *p*-value < 0.01, ns: non-significant.

Activation of CD4+ T-cells is important to augment CTL responses and to ensure the development of memory CTLs against a specific antigen. Therefore, to evaluate the helper T-cell responses induced by the Tn-PV-PS A1 (I) vaccine, CD4+ T-cell proliferation assays were performed. Initially we analyzed group A CD4+ T-cell proliferation by incubating isolated CD4+ T-cells with irradiated C57BL/6 bone marrow-derived dendritic cells (DCs) pulsed with varying concentrations of Tn-PV (II). An increase in proliferation to (II) was observed with increasing glycopeptide concentration (Figure 2A). Afterwards, we compared the proliferation of CD4+ T-cells isolated from each group of mice by pulsing DCs with Tn-PV (II) at 40 µg/mL concentration. Cell proliferation of group A mice was double that of groups B and C (Figure 2B). This proves that, while PV (III) is a known MHCII epitope, conjugation of Tn-PV (II) to MHCII-dependent PS A1 is essential to trigger a strong immune response, as PV’s small size prevents its proper processing.

**Figure 3:**
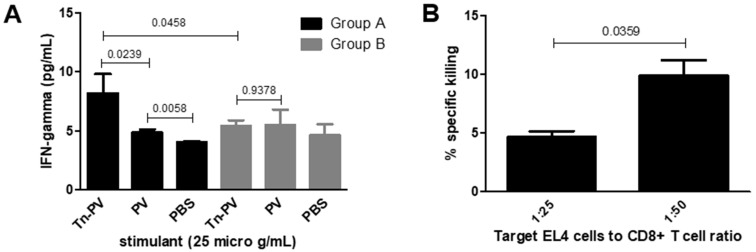
(**A**) IFN-γ production by CD8+ T-cells. BMDCs were pulsed with 25 µg/mL Tn-PV (II) or PV (III) or PBS and subsequently cultured with CD8+ T-cells from group A and group B mice with a DC-to-CD8+ T-cell ratio of 1:10 for 24 h. Supernatants were collected and IFN-γ in the supernatant was quantified by ELISA analysis. (**B**) Apoptosis of Tn-expressing tumor cell. EL4 cells were pulsed with 30 µg/mL Tn-MUC1 (IV). Pulsed and unpulsed EL4 cells were labeled with [^3^H] thymidine and then CD8+ T-cells from group A were incubated with EL4 cells with an EL4-to-CD8+ T-cell ratio of 1:25 or 1:50. Cells were harvested after 6 h and counted on a scintillation counter to determine thymidine loss by apoptotic DNA fragmentation.

CD8+ T-cell proliferation assays were performed (Figure 2C,D) by stimulating CD8+ T-cells with 10 µg/mL Tn-PV (II). Group A elicited more proliferation than group B, indicating a role of PS A1 in MHCI presentation. To confirm this unexpected result, we performed an assay to determine the production of interferon gamma (IFN-γ) by CD8+ T-cells upon incubation with antigen-pulsed DCs. Briefly, isolated CD8+ T-cells from groups A and B were cultured with DCs pulsed with either Tn-PV (II), PV (III) or PBS for 24 h at 37 °C and 5% CO_2_. Supernatants were collected for IFN-γ ELISA analysis. The assay revealed more IFN-γ production by group A compared to group B (Figure 3A) when stimulated with Tn-PV (II), and no increase when stimulated with PV peptide (III) only, which confirms that (III) remains glycosylated when binding to MHCI and targets the CD8+ immune response towards the Tn antigen. The inability of Tn-PV (II) immunization to elicit strong CD8+ proliferation and IFN-γ production on its own indicates that PS A1 plays a key role as a carrier. In APCs, PS A1 is endocytosed and processed for binding to MHCII. Similarly, the size and zwitterionic character of PS A1 could facilitate its binding to MHCI, or its cross-presentation in APCs, facilitating the delivery of the MHCI PV (III) epitope.

Finally, to test the ability of CD8+ T cells to recognize the human epitope of Tn on MUC1, we tested the lytic activity of isolated CD8+ towards Tn-negative cancer cells pulsed with Tn-MUC1. Apoptotic death is generally associated with DNA fragmentation; therefore, we performed a JAM assay [29] to measure the amount of intact DNA (live cells) present after exposure of Tn-expressing cancer cells to vaccine-primed CTLs. Tn-negative murine lymphoma EL4 cells were incubated overnight with Tn-PV (II) or Tn-MUC1 (IV) for MHCI presentation, then labeled with [^3^H] thymidine for 5 h. The cells were washed and cultured with group A CTLs or T-cell medium only using target cell-to-effector cell ratios of 1:25 and 1:50. CD8+ T-cells showed negligible specific cytotoxicity towards EL4 cells incubated with Tn-PV (II) but showed detectable specific cytotoxicity towards EL4 cells incubated with Tn-MUC1 (IV) (Figure 3B). This could be due to a lack of antigen uptake by cells incubated with Tn-PV (II). Tn-PV (II) contains 13 amino acids; more than the typical 9–10 amino acid MHCI epitope. This can hinder the presentation of the antigen on the tumor cell surface and prevent cell recognition by CTLs. CD8+ T-cells responded to the same 13 amino acid glycopeptide in the interferon release assay. The longer incubation period in the interferon assay may allow antigen processing by DCs to cross-present peptides on MHC class I. The cytotoxicity assay has a shorter incubation time with non-professional APCs. Tn-MUC1 (IV) is only 10 amino acids long and is more efficiently complexed with MHC I molecules [35]. Notably, isolated CD8+ T-cells recognized Tn-antigen bound to a peptide that is different than the immunizing peptide, and similar to its normal presentation on human cancer cells. This implies that the CTL response generated by Tn-PV-PS A1 (I) immunization is specific towards the Tn antigen. Additionally, the incapacity of Tn-PV (II) to be processed on its own indicates that a carrier is required for its proper processing by MHCI. This would explain the absence of competent CTLs after immunization with Tn-PV alone (Figure 3A).

## 4. Conclusions

To address the MHCI independence of the PS A1 zwitterionic polysaccharide, we have developed a modified PV-PS A1 carrier containing the PV peptide, predicted in silico to bind to MHCI. To test the carrier’s ability to elicit CTLs, we synthesized a Tn-PV-PS A1 vaccine (I) by solid phase synthesis targeting the Thomsen-nouveau TACA. The vaccine (I) and Tn-PV (II) were injected in different groups of mice, and T-cells were isolated. Through proliferation assays, we proved that only CD4+ T-cells isolated from mice treated with (I) could recognize Tn-PV, confirming the well-known role of PS A1 as an MHCII-dependent carrier. As predicted by computational analysis, PV did bind to MHCI, and CD8+ T lymphocytes from both groups recognized Tn-PV. However, Tn-PV-PS A1 (I) elicited a stronger response than Tn-PV (II) alone. This surprising result, confirmed by IFN-γ production assay, could result from the small size of Tn-PV (II), too small to be properly processed by MHCI. The addition of the large 110 kDa PS A1 could facilitate the vaccine’s endocytosis for MHCI presentation, similarly to PS A1’s processing by MHCII. This mechanism must still be confirmed experimentally. Finally, the resulting CD8+ T-cells can recognize and kill murine EL4 tumor cells expressing the Tn antigen bound to its biological substrate MUC1. Overall, this study (i) cements PV-PS A1 as a robust MHC-I- and MHC-II-dependent carrier, capable of eliciting both T helpers and cytotoxic T lymphocytes towards a given antigen, (ii) demonstrates the first use of a zwitterionic polysaccharide to elicit an MHCI response and (iii) proves the ability of the PV peptide, a well-known MHCII epitope, to elicit a robust CD8+ response. These promising results pave the way for a future clinical evaluation of a PV-PS A1-based vaccine, and work to ensure its wide applicability, safety and optimal efficacy is planned.

## Figures and Tables

**Table 1:** Predictor model of binding strength of PV and Chow peptides to (**A**) murine and (**B**) human MHCI molecules. Epitopes from PV peptide are noted in blue and Chow peptide in red. Used consensus/SMM method.

A	Allele	Epitope	IC_50_ (nM)	B	Allele	Epitope	IC_50_ (nM)
	H2-K^d^	TYKDTVQLRR	2913		HLA-C*03:03	KLFAVWKITYKDT	2.7
	H2-K^d^	TYKDTVQLRR	3021		HLA-B*15:01	KLFAVWKITYKDT	6.7
	H2-K^d^	KLFAVWKITYKDT	571		HLA-C*12:03	KLFAVWKITYKDT	7.5
	H2-K^d^	KLFAVWKITYKDT	3235		HLA-C*12:03	TYKDTVQLRR	7.5
	H2-K^d^	KLFAVWKITYKDT	4059		HLA-C*14:02	TYKDTVQLRR	10
	H2-K^b^	KLFAVWKITYKDT	1937		HLA-C*12:03	TYKDTVQLRR	10
	H2-K^b^	KLFAVWKITYKDT	3149		HLA-C*12:03	KLFAVWKITYKDT	17
	H2-D^b^	KLFAVWKITYKDT	3041		HLA-C*05:01	TYKDTVQLRR	25
	H2-D^b^	KLFAVWKITYKDT	4165		HLA-C*12:03	KLFAVWKITYKDT	27
					HLA-C*03:03	KLFAVWKITYKDT	29

## Data Availability

The MHC datasets used for epitope binding prediction are publicly available on the immune epitope database at www.iedb.org.

## Supplementary Materials

NMR spectra and detailed synthetic information can be downloaded at: https://www.mdpi.com/article/10.3390/vaccines12121375/s1. Scheme S1. Synthesis of Tn antigen for peptide incorporation. Scheme S2. Synthesis of Tn-MUC1 (E) by SPPS protocol.
