# Supplementary material for: The Glycopeptide PV-PS A1 Immunogen Elicits Both CD4+ and CD8+ Responses
Source: Vaccines (Basel). 2024 Dec 6;12(12):1375. doi: 10.3390/vaccines12121375 (PMC11680115; doi:10.3390/vaccines12121375)

The glycopeptide PV-PS A1 immunogen elicits both CD4+ and CD8+ responses

Sharmeen Nishat [1], Md Kamal Hossain [2], Geraud Valentin [1], Farzana Hossain [1], Gamage Shanika [1], Katherine A. Wall [2] and Peter R. Andreana [1,*]

[1]Department of Chemistry and Biochemistry and School of Green Chemistry and Engineering, The University of Toledo, 2801 West Bancroft Street, Toledo, Ohio 43606, United States.
[2]Department of Medicinal and Biological Chemistry, College of Pharmacy and Pharmaceutical Sciences, The University of Toledo, 2801 West Bancroft Street, Toledo, Ohio 43606, United States.

Corresponding Author:
*peter.andreana@utoledo.edu

Experimental Section

Materials and Methods

All reagents and solvents were purchased from Aldrich, AK Scientific, Fisher Scientific, Chem Impex and EMD Millipore. All the solvents and reagents were used without further purification, unless otherwise stated. Molecular sieves 3 Å were purchased from Aldrich and were activated by placing into a high temperature oven containing vacuum capability and an inert gas line. The sieves were heated over 150 °C in the oven under vacuum for overnight, then cooled to room temperature under inert atmospheric nitrogen. Thin layer chromatography (TLC) was performed on 0.25 mm Dynamic Adsorbents, L.L.C. pre-coated silica gel (particle size 0.03-0.07 mm, catalog no. 84111, lot # LA2006) to monitor the progress of reaction. TLC plates were visualized using UV light or by staining with *p*-anisaldehyde or ninhydrin solution followed by charring. Whatman Purasil 60 Å (230-400 mesh ASTM) silica gels were used for normal phase column chromatography. ^{1}H and ^{13}C NMR, DEPT135, HMQC, COSY spectra were acquired using Bruker 600 MHz spectrometers. The residual $CDCl_3$ singlet at δ 7.27 ppm (^{1}H NMR) and residual triplet at δ 77.23 ppm (^{13}C NMR) were used as the standard for ^{1}H NMR and ^{13}C NMR spectra respectively. The residual HDO was referenced to 4.79 when spectra was taken in D_2O Signal patterns are indicated as s: singlet; d: doublet; t: triplet; q: quartet; m: multiplet; dd: doublet of doublets; br: broad and coupling constants are reported in hertz (Hz). Low resolution mass spectra (LRMS) were recorded on an Esquire-LC electrospray ionization (ESI) mass spectrometer. High resolution mass spectra (HRMS) were achieved using a Waters Synapt High Definition. CD4+ and CD8+ T cell isolation kit was procured from Invitrogen. Male C57BL/6 mice were

obtained from Jackson Laboratory and maintained by the Department of Laboratory Animal Resources (DLAR) at the University of Toledo main campus. All animal protocols were approved by the Institutional Animal Care and Use Committee (IACUC) of the University of Toledo and experiments were performed according to the institutional guidelines.

Scheme S1. Synthesis of Tn antigen for peptide incorporation.

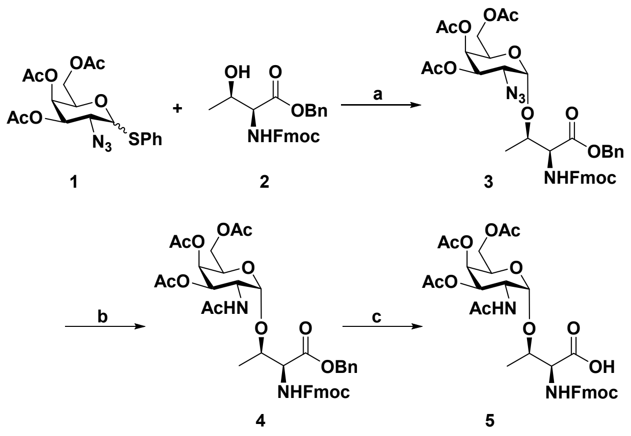

Reagents and conditions: a) NIS/TMSOTf, anhyd. DCM, MS-4Å, 0 °C to rt, 5 h, 55%; b) Zn dust, THF: AcOH: Ac_2O (3: 2: 1), rt, 5 h, 80%; c) 5% Pd-C, H_2, MeOH, 3-4 drops 1 N HCl, rt, 1 h; 72%

Synthesis of *N*-Fmoc-*O*-(2-azido-3,4,6-tri-*O*-acetyl-2-deoxy-α-D-galactopyranosyl)-L-threonine benzyl ester (3)

Glycosyl donor **1** (2 g, 4.73 mmol) and *N*-Fmoc amino acid benzyl ester acceptor **2** (1.8 g, 4.26 mmol) were dissolved in anhydrous DCM (5 mL/mmol) and then activated molecular sieves 3 Å (0.1g/1 gram of donor) were added. The reaction mixture was stirred at room temperature for 10 minutes under an atmosphere of argon, then cooled to 0 °C. To this mixture *N*-iodosuccinimide (1.5 eq) and a catalytic amount of TMSOTf (75µL/1.5 mmol of donor) were added and afterwards, the reaction was stirred at room temperature. The reaction progress was monitored by TLC

analysis. Upon completion, as noted by the TLC, the reaction mixture was filtered through Celite®-545. To the filtrate, saturated $NaHCO_3$ (5 mL), 10% sodium thiosulfate solution (5 mL) and water were added and the entire mixture was extracted with CH_2Cl_2 (3X10 mL). The organic layers were combined and washed with a saturated NaCl solution and then dried over anhydrous Na_2SO_4. Removal of solvent was carried out under reduced pressure. The crude reaction mixture was subjected to flash column chromatography for final product purification using 40% EtOAc in hexane as eluent. The compound was obtained as a pale yellow solid (1.94 g, 2.60 mmol, 55%).

^{1}H NMR (600 MHz, $CDCl_3$): δ 7.78 (d, *J* = 7.7 Hz, 2 H, Ar H), 7.63 (d, *J* = 7.7 Hz, 2 H, Ar H), 7.42-7.31 (m, 9 H, Ar H), 5.69 (d, *J* = 9.4 Hz, 1 H, -N<u>H</u>), 5.44 (d, *J* = 2.5 Hz, 1 H, H-4), 5.29-5.20 (m, 3 H, H-3, -OC<u>H</u>$_2$Ph), 4.90 (d, *J* = 3.6 Hz, 1 H, H-1), 4.50-4.47 (m, 2 H, CH_3-C<u>H</u>-, CH_3-CH-C<u>H</u>-), 4.45-4.42 (m, 1 H, H-6), 4.37-4.33 (m, 1 H, H-6′), 4.26 (t, *J* = 7.4 Hz, 1 H, H-5), 4.23 (t, *J* = 6.5 Hz, 1 H, -CH_2-C<u>H</u>-), 4.08 (d, *J* = 6.7 Hz, 2 H, -C<u>H$_2$</u>-CH-), 3.59 (dd, *J* = 3.9, 11.42 Hz, 1 H, H-2), 2.16 (s, 3 H, -COC<u>H$_3$</u>), 2.08 (s, 3 H, -COC<u>H$_3$</u>), 2.05 (s, 3 H, -COC<u>H$_3$</u>), 1.35 (d, *J* = 6.4 Hz, 3 H, -CHC<u>H$_3$</u>).

^{13}C NMR (150 MHz, $CDCl_3$): δ 170.71, 170.36, 170.22, 157.16, 144.21, 144.08, 141.62, 141.59, 135.27, 129.04, 128.96, 128.89, 128.06, 127.48, 125.61, 125.59, 120.31, 99.58, 68.58, 68.10, 67.96, 67.78, 67.37, 62.12, 59.12, 58.04, 47.42, 21.04, 20.99 (2C), 18.91; EIMS [$(M+Na)^+$] calculated for $C_{38}H_{40}N_4NaO_{12}$ is 767.2 found 767.4.

Synthesis of *N*-Fmoc-*O*-[2-(acetylamino)-3,4,6-tri-*O*-acetyl-2-deoxy-α-D-galactopyranosyl]4-L-threonine benzyl ester (4)

The glycosylated azido compound **3** (1.8 g, 2.37 mmol), zinc dust (2 w/w eq), acetic acid (15 mL/g), and acetic anhydride (10 mL/g) were dissolved in anhydrous THF (30 mL/g) under inert atmosphere and stirred at room temperature. Upon completion of the reaction (~ 3 h), the

reaction mixture was filtered through Celite®-545 and washed with excess DCM. The acid was quenched by slowly adding saturated $NaHCO_3$ and subsequently the reaction mixture was extracted with ethyl acetate (3 x 20 mL). The organic layers were combined and the solvent was removed under reduced pressure. Flash chromatography purification using 70% EtOAc in hexane resulted pure compound **4**. The compound was obtained as a pale yellow solid (1.47 g, 1.93 mmol, 80%).

^{1}H NMR (600 MHz, $CDCl_3$): δ 7.80 (d, *J* = 7.8 Hz, 2 H, Ar H), 7.65 (d, *J* = 7.4 Hz, 2 H, Ar H), 7.44-7.33 (m, 9 H, Ar H), 5.73 (d, *J* = 10.1 Hz, 1 H, -N$\underline{H}$Ac), 5.53 (d, *J* = 9.6 Hz, 1 H, -N$\underline{H}$Fmoc), 5.39 (d, *J* = 2.3 Hz, 1 H, H-4), 5.21 (d, *J* = 11.7 Hz, 1 H, -OC$\underline{H}_2$Ph), 5.10-5.07 (m, 2 H, H-3, -OC$\underline{H}_2$Ph), 4.81 (d, *J* = 3.8 Hz, 1 H, H-1), 4.57-4.53 (m, 1 H, H-2), 4.49-4.44 (m, 3 H, -C$\underline{H}_2$-CH-, CH_3-CH-C$\underline{H}$-), 4.28 (t, 1 H, *J* = 7.1 Hz, -CH_2-C$\underline{H}$-), 4.24 (m, 1 H, CH_3-C$\underline{H}$-), 4.21 (t, *J* = 6.4 Hz, 1 H, H-5), 4.13-4.06 (m, 2 H, H-6, H-6′), 2.18 (s, 3 H, -COC$\underline{H}_3$), 2.05 (s, 3 H, -COC$\underline{H}_3$), 2.03 (s, 3 H, -COC$\underline{H}_3$), 2.00 (s, 3 H, -COC$\underline{H}_3$), 1.32 (d, *J* = 6.2 Hz, 3 H, -CHC$\underline{H}_3$).

^{13}C NMR (150 MHz, $CDCl_3$): δ 171.30, 171.07, 170.70, 170.66, 170.64, 156.81, 144.08, 143.98, 141.64, 134.68, 129.31, 129.22, 128.93, 128.89, 128.12, 127.47, 125.45, 125.40, 120.39, 120.36, 100.27, 68.77, 68.18, 67.78, 67.63, 67.58, 62.45, 58.86, 47.83, 47.47, 23.56, 21.12, 21.09, 20.98, 18.50; EIMS [$(M+Na)^+$] calculated for $C_{40}H_{44}N_2NaO_{13}$ is 783.3 found 783.5.

Synthesis of *N*-Fmoc-*O*-[2-(acetylamino)-3,4,6-tri-*O*-acetyl-2-deoxy-α-D-galactopyranosyl]-L-threonine carboxylic acid (5)

Compound **4** (0.5 g, 0.66 mmol) was dissolved in methanol and 5% Pd-C (100 mg) and 3-4 drops 1 N HCl were added to the solution. The entire reaction mixture was subjected to hydrogenolysis in 35 psi hydrogen pressure using Purr apparatus. After 1 hour, the reaction was stopped and the reaction mixture was filtered through Celite®-545 bed and then washed with

DCM. Combined filtrate was evaporated under reduced pressure. The unreacted starting material was recovered from the reaction mixture by flash chromatography using 70% EtOAc in hexane. Afterwards flash chromatography elution of the compound with 6% methanol in DCM followed by evaporation of solvent resulted compound **5** as a white solid (0.32 g, 0.48 mmol, 72%).

EIMS [$(M+Na)^+$] calculated for $C_{33}H_{38}N_2NaO_{13}$ is 693.2 found 693.1.

^{1}H NMR (600 MHz, $CDCl_3$): δ 7.78-7.73 (q, J_1 = 6.6 Hz, J_2 = 8.0 Hz, 2 H, Ar H), 7.63-7.50 (d, *J* = 8.0 Hz, 2 H, Ar H), 7.42-7.37 (m, 2 H, Ar H), 7.33-7.29 (m, 2 H, Ar H), 6.25-6.23 (d, *J* = 9.3 Hz, 1 H), 6.03-6.02 (d, *J* = 8.8 Hz, 1 H), 5.39-5.38 (d, *J* = 2.0 Hz, 1 H), 5.29-5.26 (m, 1 H), 5.17-5.14 (dd, *J* = 3.4 Hz, 1 H), 5.05-5.04 (d, *J* = 2.9 Hz, 1 H), 4.66-4.63 (m, 1 H), 4.58-4.55 (m, 1 H), 4.51-4.42 (m, 4 H), 4.35-4.30 (m, *J* = 3.0 Hz, 1 H), 4.27-4.24 (m, 2 H), 4.20-4.18 (t, *J* = 5.8 Hz, 1 H), 4.15-4.08 (m, *J* = 3.0 Hz , 3 H), 2.18-2.16 (d, *J* = 7.3 Hz, 3 H), 2.05 (s, 3 H), 2.02 (s, 2 H), 1.99 (m, 3 H), 1.30-1.27 (m, 3 H), 1.02-1.00 (d, *J* = 5.8 Hz, 1H).

Characterization of Tn-PV-ONH_2 (6)

The compound was obtained as an off white solid. Purity of the compound was determined as 93.28% by HPLC (A: 0.1% TFA in H2), B: acetonitrile).

^{1}H NMR (600 MHz, D_2O): δ 7.59 (d, *J* = 7.7 Hz, 1 H, Ar H), 7.45 (d, *J* = 8.1 Hz, 1 H, Ar H), 7.33-7.30 (m, 2 H, Ar H), 7.28-7.26 (m, 1 H, Ar H), 7.22-7.19 (m, 4 H, Ar H), 7.11 (t, *J* = 7.7 Hz, 1 H, Ar H), 7.07 (d, *J* = 8.6 Hz, 2 H, Ar H), 6.77 (d, *J* = 8.6 Hz, 2 H, Ar H), 4.67-4.65 (m, 2 H), 4.62-4.60 (m, 1 H), 4.56-4.53 (m, 1 H), 4.46-4.45 (m, 1 H), 4.38-4.37 (m, 3 H), 4.35 (dd, *J* = 3.0, 6.38 Hz, 1 H), 4.29-4.27 (m, 2 H), 4.25 (t, *J* = 7.1 Hz, 1 H), 4.20-4.18 (m, 3 H), 4.11-4.09 (m, 1 H), 4.04 (dd, *J* = 3.9, 11.1 Hz, 1 H, H-2), 3.98 (d, *J* = 7.4 Hz, 1 H), 3.94-3.92 (m, 1 H, H-5), 3.89 (d, *J* = 2.3 Hz, 1 H, H-4), 3.83 (dd, *J* = 3.2, 10.98 Hz, 1 H, H-3), 3.72-3.65 (m, 2 H, H-6, H-6′),

3.26 (dd, J = 6.9, 14.80 Hz, 1 H), 3.20 (dd, J = 7.2, 14.80 Hz, 1 H), 3.10-3.07 (m, 1 H), 3.06-3.02 (m, 1 H), 2.98-2.95 (m, 3 H), 2.93-2.91 (m, 3 H), 2.88-2.84 (m, 3 H), 2.77-2.73 (m, 1 H), 2.69 (s, 1 H), 1.98 (s, 3 H), 1.76-1.62 (m, 10 H), 1.57-1.50 (m, 5 H), 1.44-1.29 (m, 6 H), 1.18-1.15 (m, 11 H), 0.89-0.83 (m, 11 H), 0.81 (d, J = 6.3 Hz, 3 H), 0.74 (d, J = 6.7 Hz, 3 H).

^{13}C NMR (150 MHz, D_2O): δ 182.70, 178.33, 174.00, 173.97, 173.44, 172.97, 172.31, 171.13, 163.09, 162.86, 154.45, 145.38, 136.13, 130.46, 129.11, 128.64, 127.89, 127.07, 124.26, 119.31, 117.23, 115.28, 111.82, 108.51, 99.08, 76.83, 72.56, 71.29, 68.45, 67.29, 61.24, 58.51, 54.64, 53.27, 52.18, 50.22, 39.58, 39.06, 38.63, 36.85, 35.49, 30.69, 30.56, 30.30, 26.32, 26.19, 24.17, 22.24, 22.02, 21.94, 21.89, 21.83, 20.68, 18.87, 18.26, 18.01, 17.74, 16.40, 14.55, 9.94; HRMS: TOF [$(M+2H)^+/2$] calculated for $(C_{89}H_{140}N_{19}O_{26})/2$ is 945.5108, found 945.5108.

Characterization of Tn-PV (II)

The compound was obtained as an off-white solid. Purity of the compound was determined as 96.65% by HPLC (A: 0.1% TFA in H2), B: acetonitrile).

^{1}H NMR (600 MHz, D_2O): δ 7.59 (d, J = 7.7 Hz, 1 H, Ar H), 7.45 (d, J = 8.1 Hz, 1 H, Ar H), 7.33-7.31 (m, 2 H, Ar H), 7.28-7.26 (m, 1 H, Ar H), 7.23-7.19 (m, 4 H, Ar H), 7.13-7.11 (m, 1 H, Ar H), 7.08 (d, J = 8.3 Hz, 2 H, Ar H), 6.77 (d, J = 8.3 Hz, 2 H, Ar H), 4.74-4.72 (m, 1 H), 4.66-4.64 (m, 1 H), 4.63-4.60 (m, 1 H), 4.56-4.54 (m, 1 H), 4.46-4.45 (m, 1 H), 4.37-4.35 (m, 1 H), 4.29-4.24 (m, 2 H), 4.22-4.16 (m, 4 H), 4.09-4.08 (m, 1 H), 4.06-4.03 (m, 1 H), 3.99 (d, J = 7.9 Hz, 1 H), 3.93-3.91 (m, 2 H), 3.89 (s, 1 H), 3.83-3.80 (m, 1 H), 3.72-3.65 (m, 2 H), 3.26-3.18 (m, 1 H), 3.09-3.03 (m, 2 H), 2.99-2.94 (m, 3 H), 2.91-2.87 (m, 5 H), 2.82-2.76 (m, 2 H), 2.20 (s, 3 H), 1.97 (s, 3 H), 1.80-1.76 (m, 3 H), 1.75-1.71 (m, 2 H), 1.69-1.60 (m, 5 H), 1.57-1.49 (m, 5 H), 1.44-1.36 (m, 3 H), 1.31-1.25 (m, 2 H), 1.20-1.14 (m, 11 H), 0.88 (d, J = 6.2 Hz, 5 H), 0.86(m,

9 H), 0.74 (d, J = 6.8 Hz, 3 H); EIMS [(M+H)$^+$] calculated for $C_{87}H_{136}N_{18}O_{24}$ is 1816.9, found 1816.5.

Characterization of PV (III)

The compound was obtained as a white solid. Purity of the compound was determined as 96.65% by HPLC (A: 0.1% TFA in H2), B: acetonitrile).

^{1}H NMR (600 MHz, D_2O): δ 7.59-7.57 (m, 1 H), 7.46-7.44 (m, 1 H), 7.32-7.29 (m, 2 H), 7.28-7.25 (m, 1 H), 7.23-7.17 (m, 4 H), 7.12-7.09 (m, 1 H), 7.08-7.06 (m, 2 H), 6.79-6.77 (m, 2 H), 4.76-4.72 (m, 1 H), 4.65-4.62 (m, 1 H), 4.57-4.54 (m, 2 H), 4.36-4.35 (m, 1 H), 4.31-4.25 (m, 4 H), 4.22-4.18 (m, 2 H), 4.09-4.07 (m, 1 H), 4.00-3.98 (m, 2 H), 3.93-3.90 (m, 1 H), 3.21-3.20 (m, 2 H), 3.10-3.03 (m, 1 H), 3.00-2.79 (m, 10 H), 1.99-1.97 (m, 1 H), 1.78 (br s, 3 H), 1.68-1.63 (m, 6 H), 1.54-1.46 (m, 6 H), 1.39 (br s, 1 H), 1.32-1.28 (m, 4 H), 1.19-1.13 (m, 8 H), 1.10-1.08 (m, 4 H), 0.89-0.83 (m, 15 H), 0.71-0.69 (m, 3 H); EIMS: TOF [(M+Na)$^+$] calculated for $C_{79}H_{122}N_{17}NaO_{19}$ is 1633.9, found 1636.8.

Glycopeptide-PS A1 Conjugation (I)

0.001 g (9.1X10^{-9} mol) PS A1 was dissolved in 0.4 mL of 100 mM NaOAc buffer (pH 5.0) in an amber vial. To this solution 0.1 mL of 5 mM $NaIO_4$ was added to obtain overall 1 mM $NaIO_4$ concentration in the reaction mixture was stirred at room temperature in dark for 60 minutes to afford PS A1 aldehyde. The reaction mixture was purified using spin column with 3k molecular weight cut off. Nano-pure water was used to drain any residual $NaIO_4$ through the column by centrifuging it at 12000 rpm for 20 minutes and the process was repeated 4 times. Pure PS A1 aldehyde was dissolved in 100 mM NaOAc buffer (pH 5.0) and 0.0026 g glycopeptide (0.0014 mmol, 3 equiv. considering PS A1 has an average MW of 110K g/mol and 120 repeating units per mole) was added to the solution. The reaction was continued at 37 °C overnight in the dark and

after that the desired product was purified using a 10k molecular weight cut off spin column. Nano pure water was used for spin column and the washing was repeated 5 times. Following D_2O washing, ^1H NMR spectra was recorded and formation of conjugate was confirmed by observing oxime doublet at δ 7.55. The product was again subjected to spin column to remove D_2O and the volume of the product was brought to 1.5 mL with sterilized water. 0.5 mL solution was pooled out and lyophilized to get the white solid product. The lyophilized product was partially soluble in water. Therefore, the remaining part of the product was not lyophilized, instead following buffer (PBS) exchange, the product was stored at 4 °C for immunization. The weight of the product was calculated from the weight of the lyophilized product.

The % loading was calculated based on integration value of oxime protons on ^1H-NMR spectrum and compared it with methyl proton present on pyruvate ring acetal of PS A1. The loading of antigen was obtained as about 10% by using the following formula:

{MW of glycopeptide/(MW of PS A1 + MW of glycopeptide)} X (Mole fraction of oxime H) X 100,

Where mole fraction of oxime H = Oxime H integration/(methyl proton integration/3)

Compare to our previous conjugation strategy,[ref] we conducted the oxidation of PS A1 with less amount of oxidant for less period of time as our goal was to reduce the oxidation of PS A1. With more oxidized PS A1, white precipitate was observed during conjugation reaction and the precipitate was insoluble in water. We were able to overcome this problem by controlling oxidation and using excess solvent.

Synthesis of Protected Tn-MUC1 (10)

N-Fmoc-proline loaded 2-chlorotrityl resin was allowed to swell in anhydrous DCM for one hour. After that, the resin was washed two times with anhydrous DCM followed by anhydrous

DMF. 20% piperidine in DMF was added to the resin and stirred for 15 minutes to deprotect Fmoc group. Subsequently the resin was washed three times using anhydrous DMF for three times. This Fmoc deprotection step was repeated two times. Coupling of amino acids were initiated by pre-activation of *N*-Fmoc amino acids for 3 minutes. 2 equivalent amino acid was treated with 2 equivalent HATU, 2 equivalent HOAt and 4 equivalent DIPEA in minimum amount of DMF to form active ester which was then transferred to the reaction cartridge containing resin and stirred in inert atmosphere for 3-6 hours depending on the amino acid that was coupled. Coupling of Tn antigen required longer time than coupling of other amino acids. Completion of the reaction was monitored by Kaiser's ninhydrin test. After completion of the reaction, the resin was again washed with plenty of anhydrous DMF. As an extra caution, *N*-terminus capping was performed by stirring the resin with Ac_2O/ DIPEA/ DMF (4.75/ 2.25/ 93, v/v/v) in presence of 0.013 M HOBt for 4 minutes followed by washing with anhydrous DMF and the process was repeated three times. This is how coupling of 10 amino acids were performed to obtain resin bound fully protected Tn-MUC1 (9). Compound 9 was cleaved from the resin by stirring the resin with AcOH : TFE : DCM (2 : 2: 6) for 2.5 h under inert atmosphere to afford compound **10**. Subsequently, compound **10** was collected by filtration followed by washing the resin with DCM. Solvent was evaporated and AcOH was co-evaporated with n-hexane to obtain crude compound as a white solid. Flash chromatography purification using 10% MeOH in DCM as eluent provided compound **10** as a white solid.

^{1}H NMR (600 MHz, $CDCl_3$): δ 7.77 (d, *J* = 7.1 Hz, 2 H), 7.60 (d, *J* = 7.1 Hz, 2 H), 7.40 (m, 2 H), 7.32 (t, *J* = 7.3 Hz, 2 H), 7.13 (d, *J* = 7.4 Hz, 2 H), 6.82 (d, *J* = 7.5 Hz, 2 H), 5.12 (s, 2 H), 4.73-4.54 (m, 4 H), 4.41-4.35 (m, 3 H), 4.24-4.17 (m, 4 H), 4.14-4.11 (m, 5 H), 3.67-3.80 (m, 4 H), 3.36 (s, 3 H), 2.95 (s, 2 H), 2.57-2.51 (m, 12 H), 2.15 (br s, 4 H), 2.08 (br s, 5 H), 2.02-1.96

(m, 18 H), 1.63 (br s, 5 H), 1.45-1.43 (m, 18 H), 1.33-1.29 (m, 18 H), 1.19-1.17 (m, 20 H); EIMS [(M+Na)$^+$] calcd for $C_{96}H_{138}N_{14}NaO_{29}S$ is 2006.9, found 2007.1

Scheme S2. Synthesis of Tn-MUC1 (**E**) by SPPS protocol.

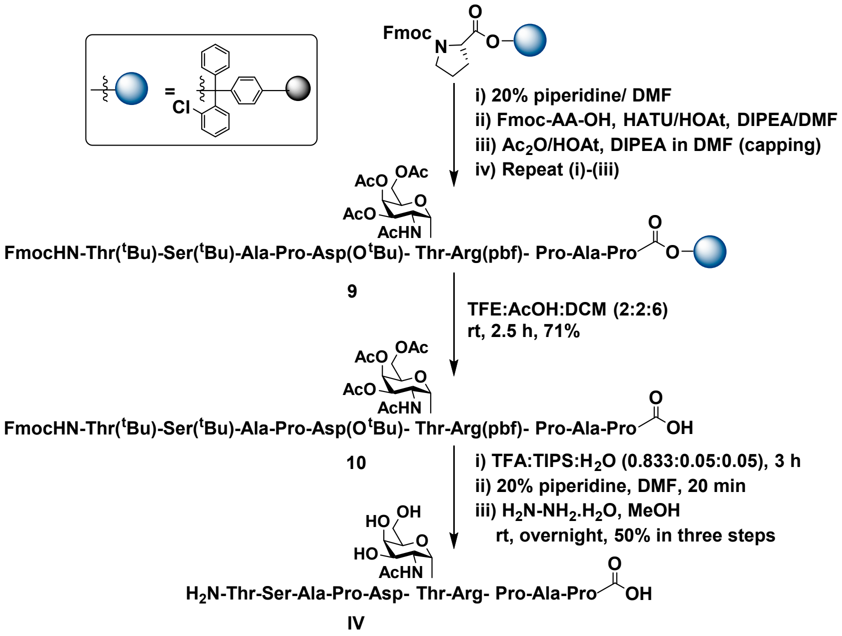

Synthesis of Tn-MUC1 (IV)

Compound **10** (9 mg, 0.005 mmol) was treated with acid cocktail (0.5 mL) composed of TFA : TIS : H_2O (0.833 : 0.05 : 0.05) and stirred for 3 h. Afterwards, the reaction mixture was added dropwise to 20 mL chilled ether (-20 °C). Immediately white precipitate was formed and

the solution was kept at -20 °C for additional 3 h for the completeness of precipitation. The precipitate was collected by filtration followed by washing with chilled ether. The precipitate was analyzed by mass spectrum to identify complete removal of acid labile protecting groups. EIMS [$(M+H)^+$] calcd for $C_{71}H_{99}N_{14}O_{26}$ is 1563.7, found. 1563.9. The precipitate was then stirred with 20% piperidine in DMF for 20 minutes under inert atmosphere to deprotect Fmoc group. Evaporation and co-evaporation of solvent with n-heptane resulted a light yellow gum which was analyzed by mass spectrum for the complete deprotection of Fmoc group. Finally acetate groups were removed by treating the Fmoc deprotected compound with hydrazine hydrate in MeOH with overnight stirring. After completion of the reaction, solvent was evaporated in reduced pressure and the crude was subjected to a P-2 Biogel column for the purification of synthesized Tn-MUC1 (**IV**) using water as eluent. The pure compound **IV** was obtained as a white solid (2.7 mg, 0.002 mmol, 50% yield in three steps).

^{1}H NMR (600 MHz, D_2O): δ 4.74-4.71 (m, 3 H), 4.63-4.61 (m, 2 H), 4.54-4.51 (m, 2 H), 4.48 (br s, 2H), 4.42-4.40 (m, 2 H), 4.37-4.35 (m, 3 H), 4.22-4.19 (m, 1 H), 4.06-4.05 (m, 2 H), 4.02-3.98 (m, 1 H), 3.95 (s, 1 H), 3.89-3.81 (m, 4 H), 3.77-3.72 (m, 5 H), 3.66-3.61 (m, 4 H), 3.20-3.19 (m, 2 H), 2.77-2.59 (m, 2 H), 2.27 (br s, 2 H), 2.21-2.16 (m, 1 H), 2.03-1.94 (m, 10 H), 1.89-1.86 (m, 5 H), 1.69-1.68 (m, 4 H), 1.35 (d, J = 6.7 Hz, 6 H), 1.29-1.28 (m, 1 H), 1.23-1.18 (m, 6 H); EIMS [$(M+H)^+$] calculated for $C_{50}H_{83}N_{14}O_{21}$ is 1215.6, found 1215.6.

^{1}H NMR of 3

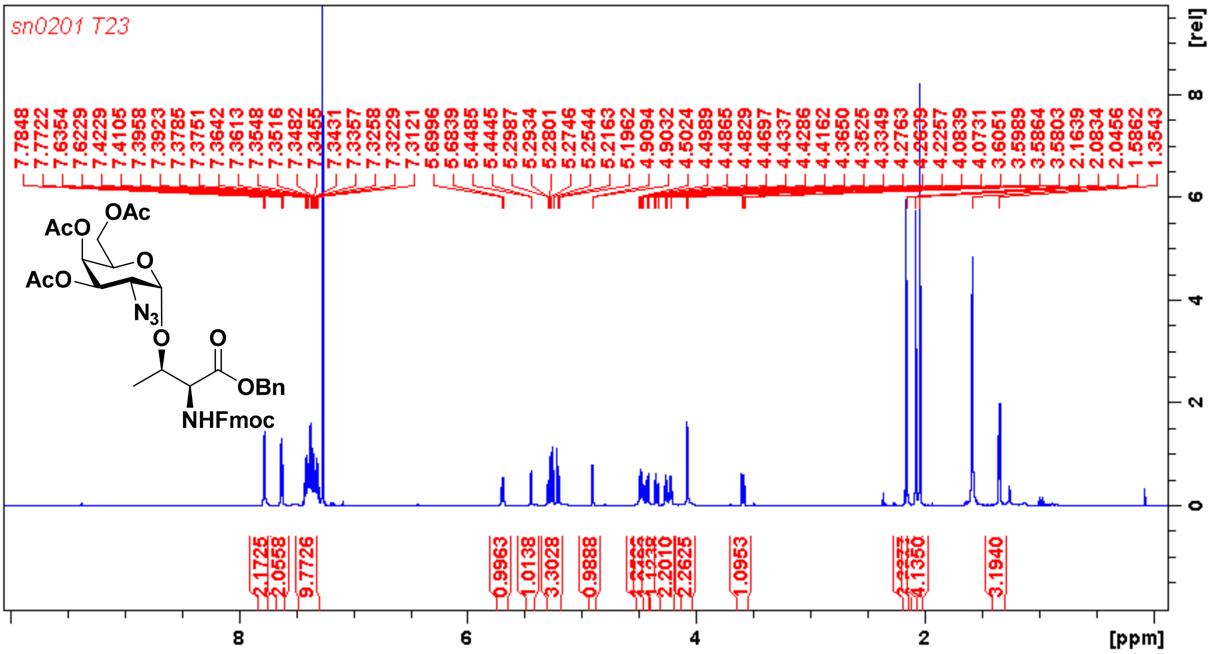

^{13}C NMR of 3

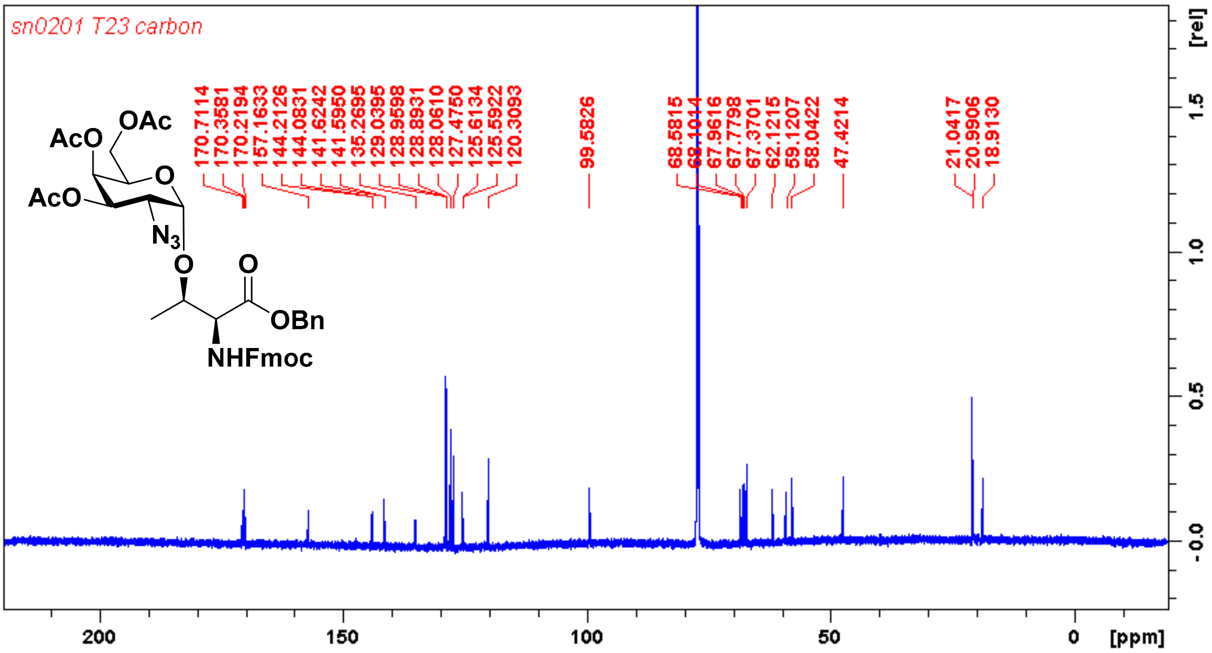

COSY of 3

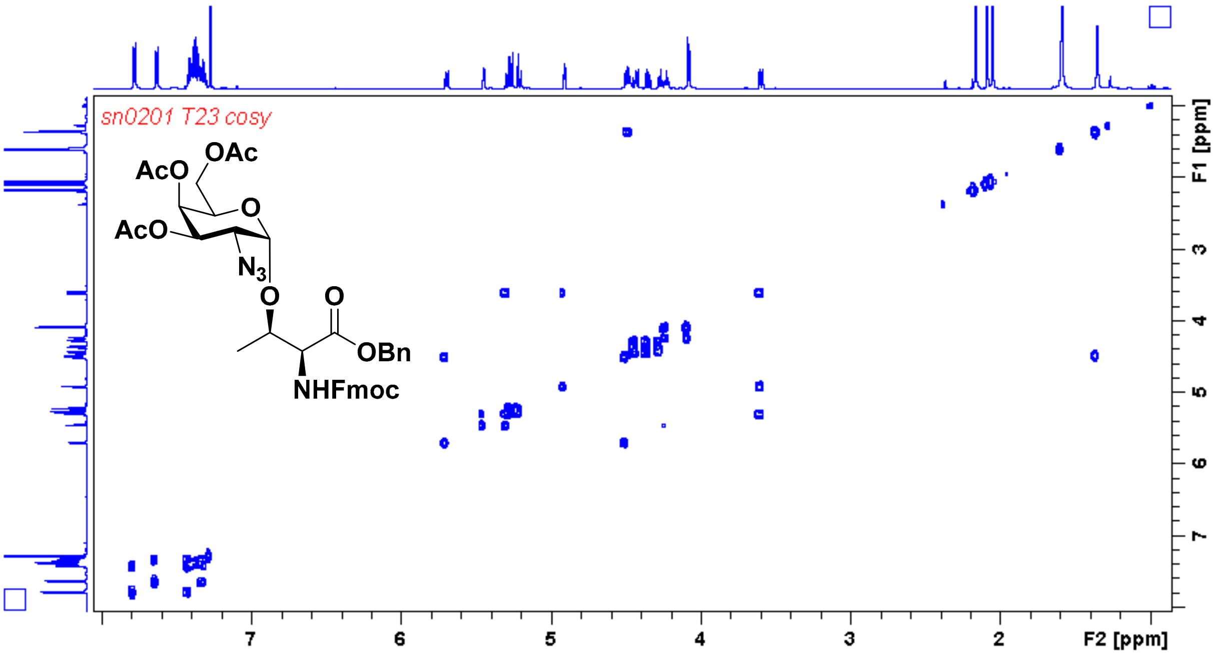

^{1}H NMR of 4

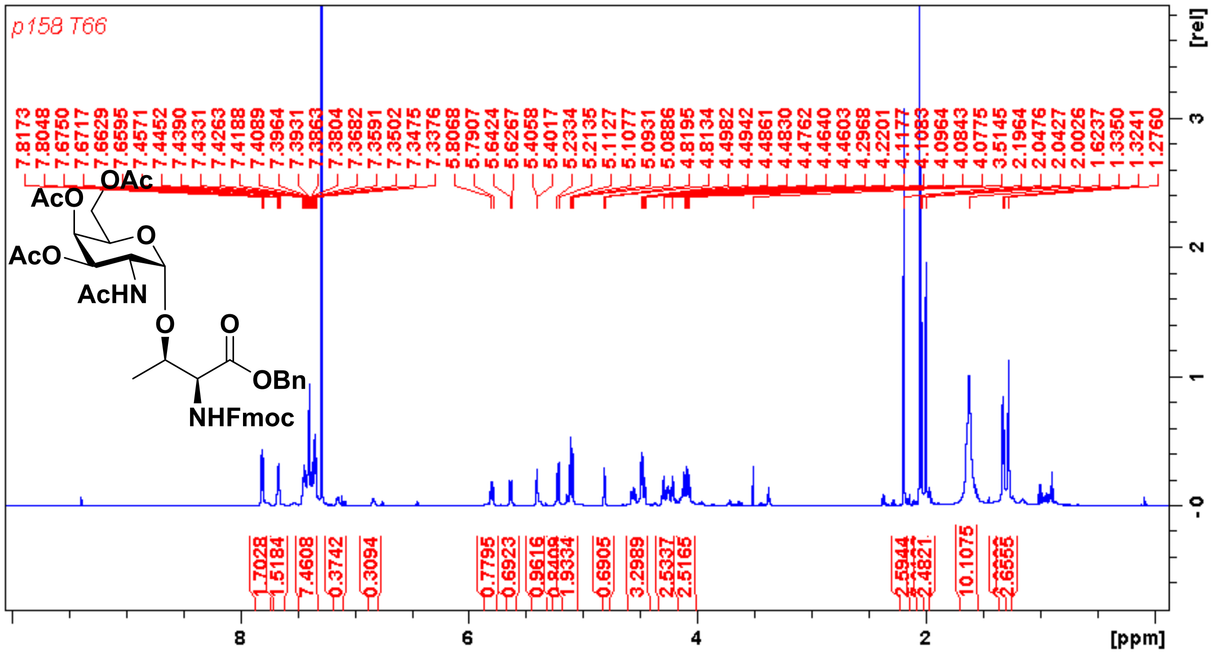

^{13}C NMR of 4

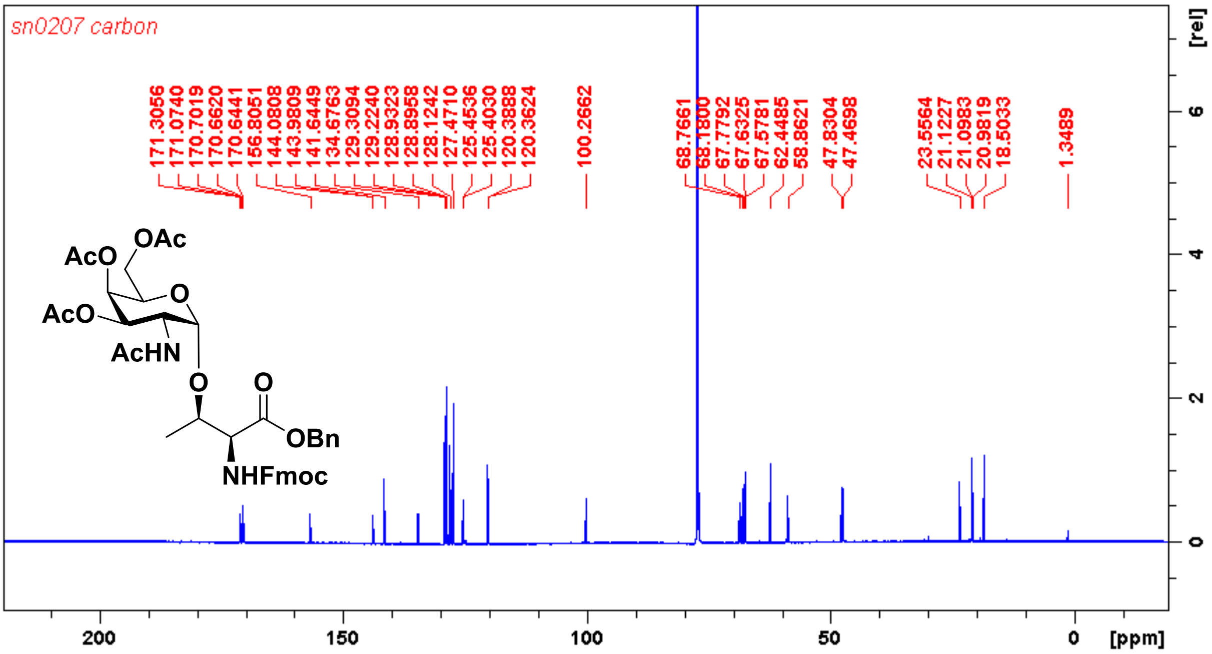

HMQC of 4

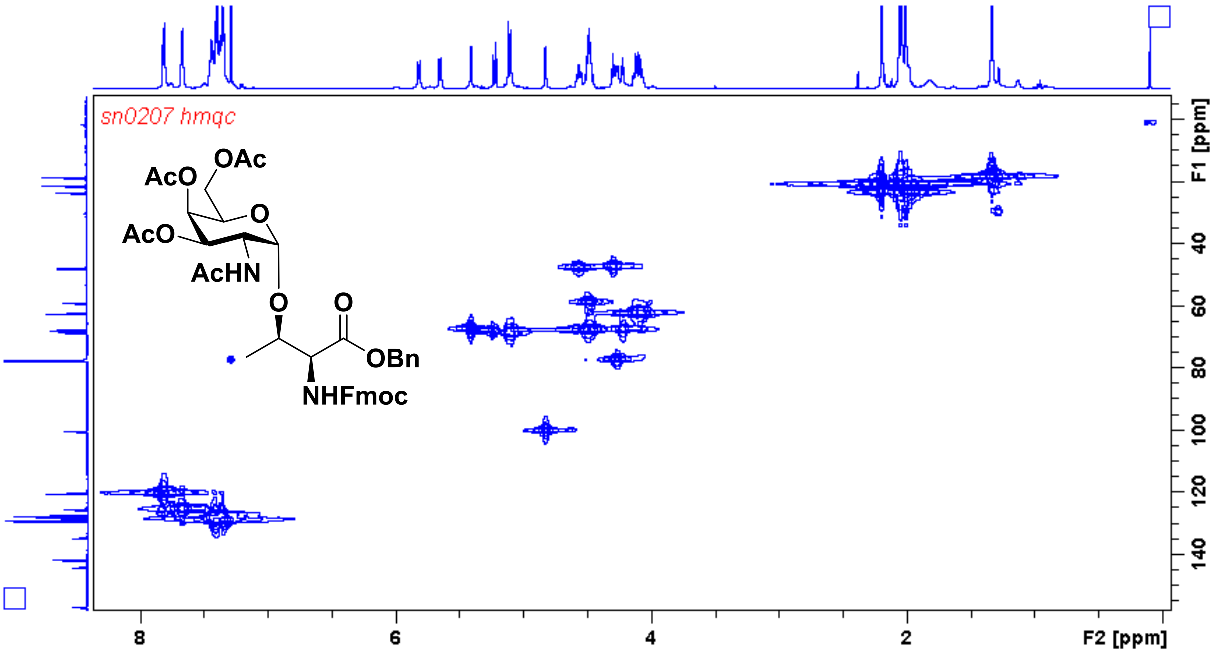

^{1}H NMR of 5

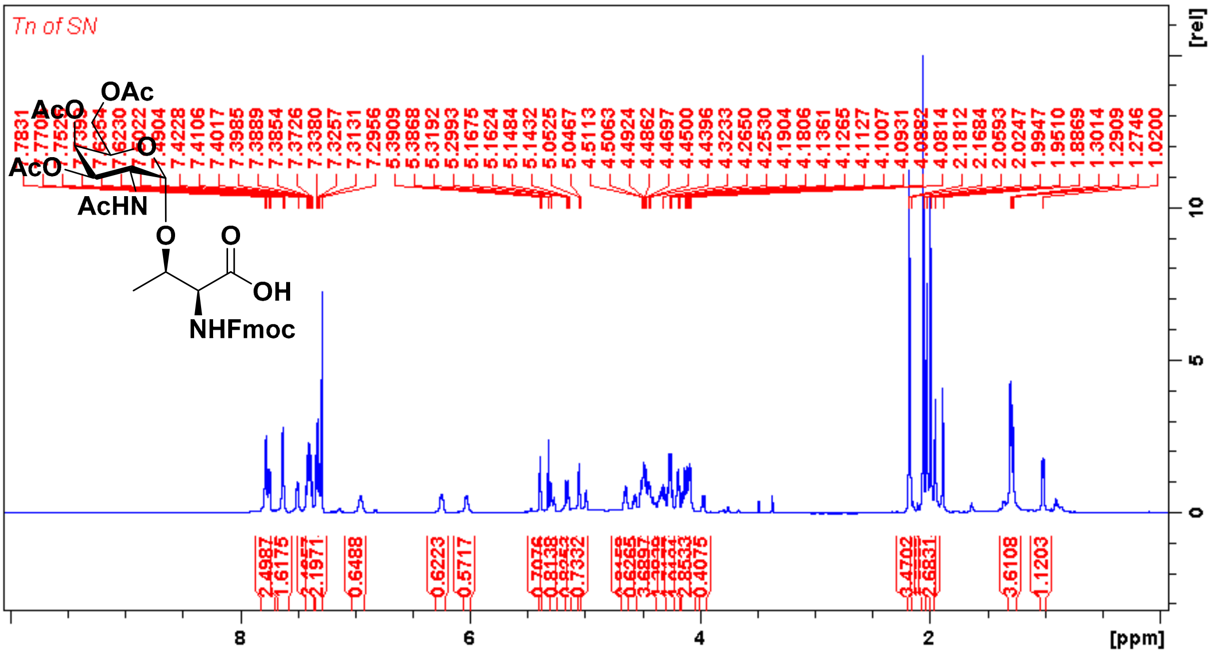

^{1}H NMR of Tn-PV-ONH$_2$ (6)

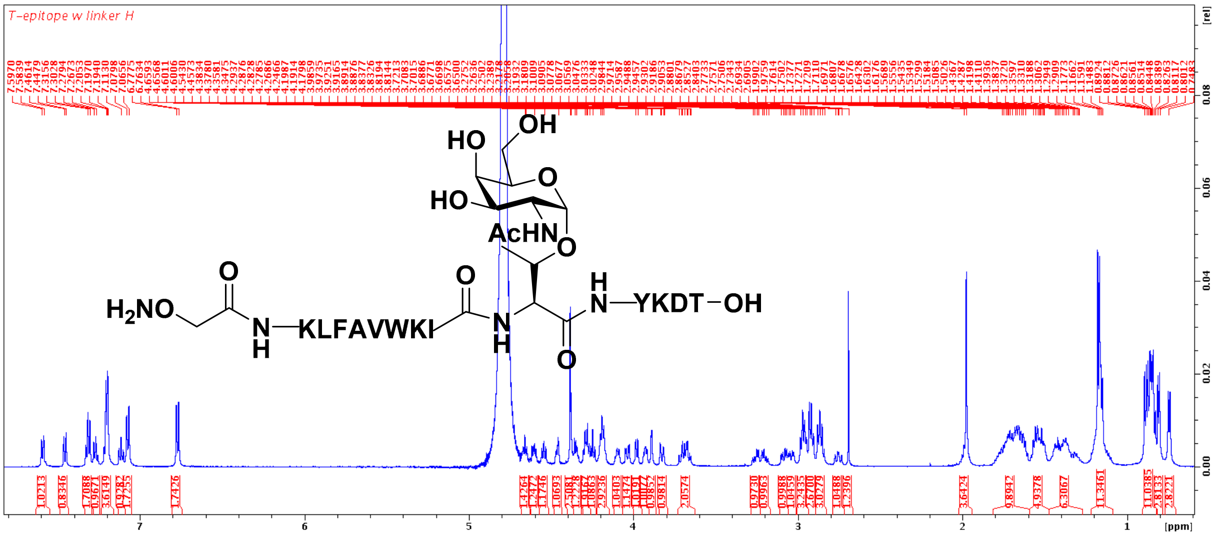

^{13}C NMR of Tn-PV-ONH$_2$ (6)

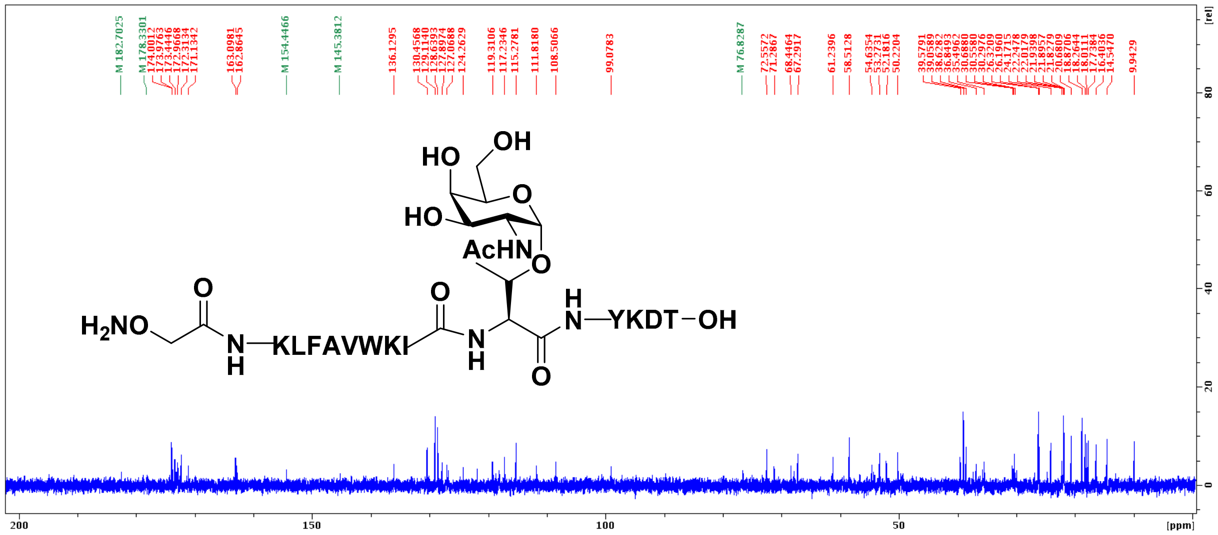

^{1}H NMR of Tn-PV (B) (7)

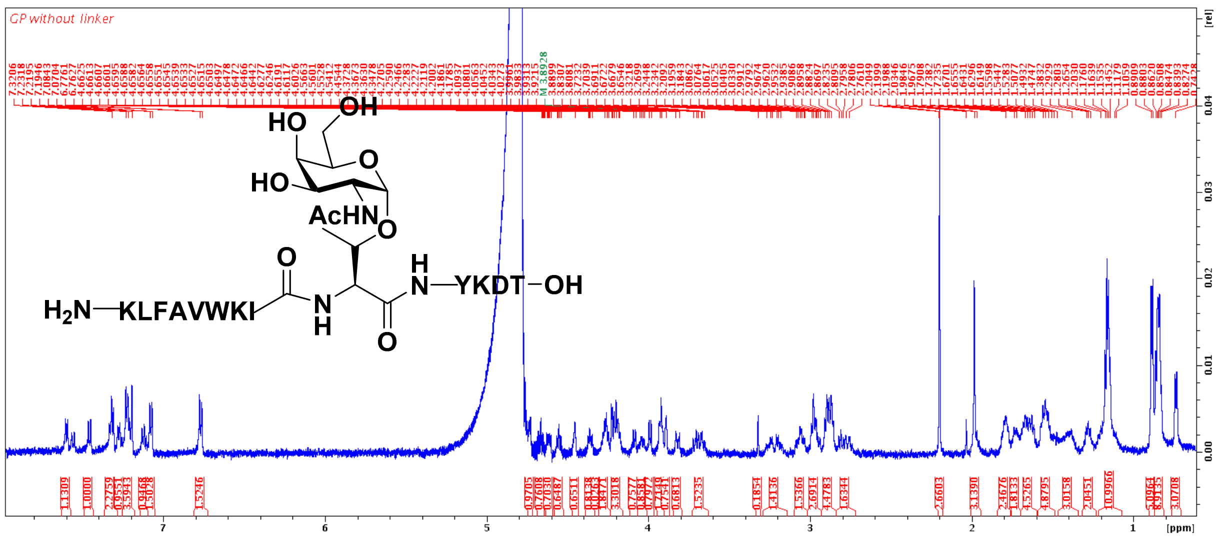

^{1}H NMR of Glycopeptide-PS A1 Conjugation (9)

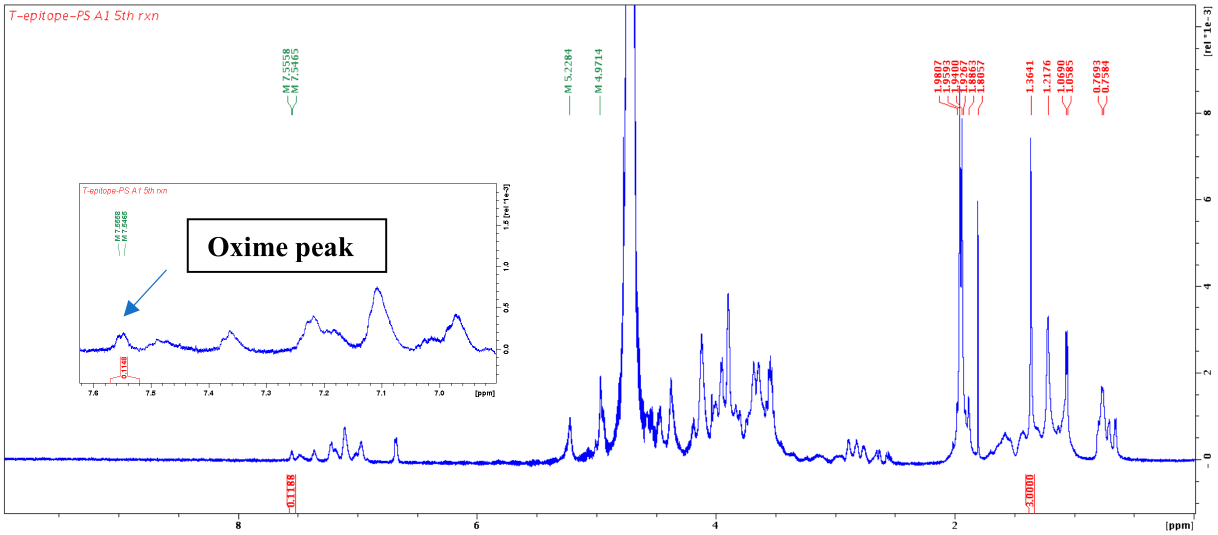

^{1}H NMR of Glycopeptide-PS A1 Conjugation comparison

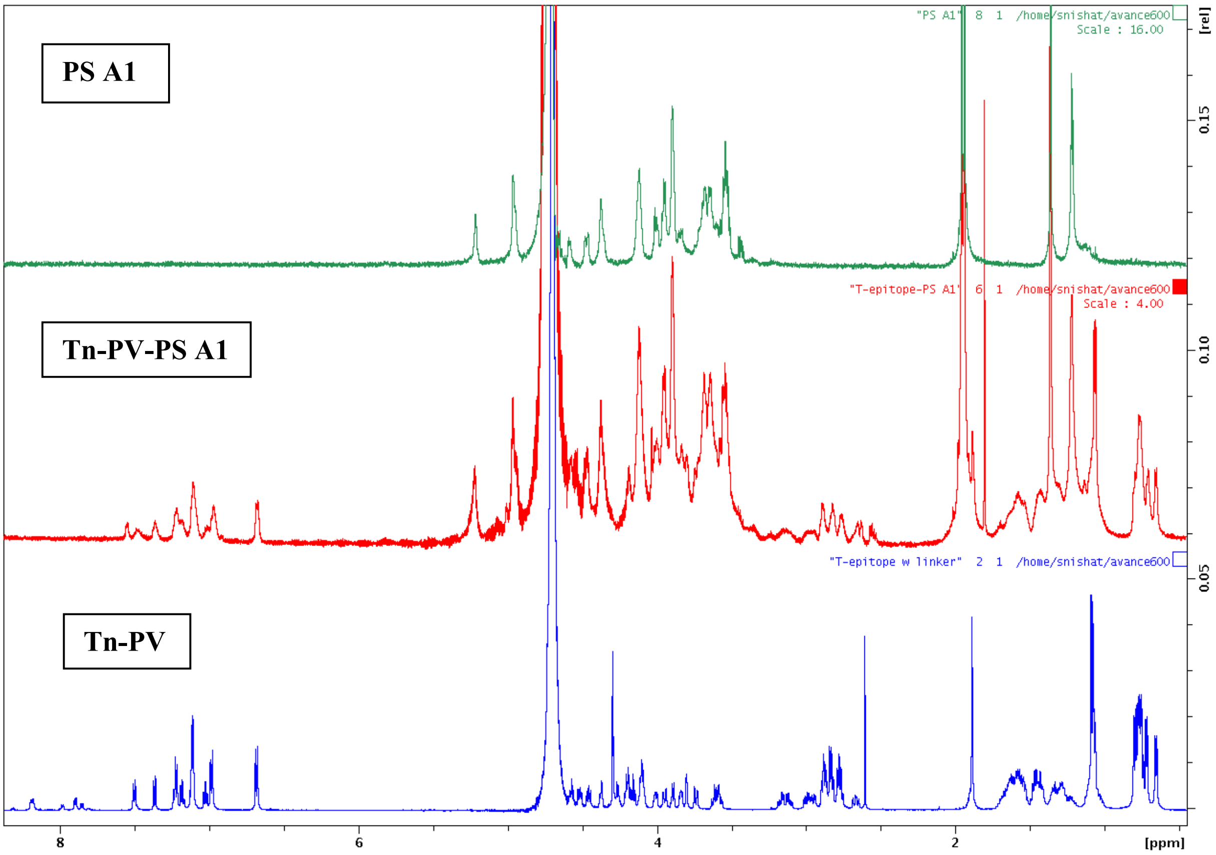

Supplement: Supplementary file 1 [file vaccines-12-01375-s001.zip › vaccines-3275253-supplementary.pdf]
